# ARTNet for Micro-Expression Recognition

**DOI:** 10.3390/s26010247

**Published:** 2025-12-31

**Authors:** Chao Wan, Wenbing Zhang, Yadong Chen, Liangliang Song, Peng Cheng

**Affiliations:** 1 Institute of Energy, Hefei Comprehensive National Science Center (Anhui Energy Laboratory), Hefei 230031, China; wanchao@ie.ah.cn (C.W.);; 2 School of Aeronautics and Astronautics, Sichuan University, Chengdu 610207, China; 3 National Key Laboratory of Fundamental Science on Synthetic Vision, Sichuan University, Chengdu 610065, China

**Keywords:** micro-expression recognition, optical flow, amplification network, transformer

## Abstract

The field of micro-expression recognition (MER) has garnered considerable attention for its potential to reveal an individual’s genuine emotional state. However, MER remains a formidable challenge, primarily due to the subtle nature and brief duration of micro-expressions. Many approaches typically rely on optical flow to capture motion between video frames. However, these methods exhibit limited variability in expression intensity across frames, which may not be effective for all individuals due to significant differences in their micro-expressions. To address this issue, we propose a novel framework called the Action Amplification Representation and Transformer Network (ARTNet) to adjust the motion amplitude, making it easier to recognize each individual’s micro-expressions. Firstly, we amplify the motion discrepancies between frames to enhance expression intensity. Subsequently, we calculate the optical flow of these amplified frames to depict micro-expressions more prominently. Finally, we use transformer layers to capture the relationships between different amplification features. Extensive experiments conducted on three diverse datasets confirm the efficacy of our proposed method.

## 1. Introduction

Facial expressions play a crucial role in discerning human emotions [1]. Therefore, capturing the state of micro-expressions promptly through automated means is highly valuable for addressing practical issues. Micro-expression recognition can be utilized to detect deceptive behavior [2], aiding in identifying falsehoods in various scenarios. This technology can assist in determining whether an interviewee or interrogated individual is concealing the truth. Compared to macro-expressions, micro-expressions have a shorter duration and lower amplitude of movement, making micro-expression recognition a challenging task. However, the study of micro-expressions continues to present many difficulties [3], such as their short duration and low range of motion. Moreover, it is challenging to determine a person’s emotional state by observing changes in a timely manner [4].

Because of the need to explore real human emotions, more and more researchers are studying how to monitor human micro-expressions. The small magnitude of micro-expressions’ movements leads to poor recognition accuracy. So, how to enhance their amplitude is a question worth investigating. Li Y et al. [5] employed a learning-based video motion magnification network to enhance facial movements in the apex frame. By employing this approach, the connections between various facial regions become more evident, thereby enhancing the effectiveness of subsequent micro-expression recognition tasks. Li et al. utilized Eulerian Motion Magnification [6] to enhance the apex frame extracted from original micro-expressions. This magnification technique effectively enhances the distinctions among various micro-expression categories, making it easier for the network to learn discriminative features. However, this amplification technique is generalized and does not effectively account for individual differences, resulting in insignificant extracted features, which may limit the performance of micro-expression recognition. In contrast, we amplify the motion amplitude of micro-expressions and utilize the optical flow method to capture the motion relationships between onset and apex frames, thereby ensuring high recognizability of micro-expressions.

**Figure 1:**
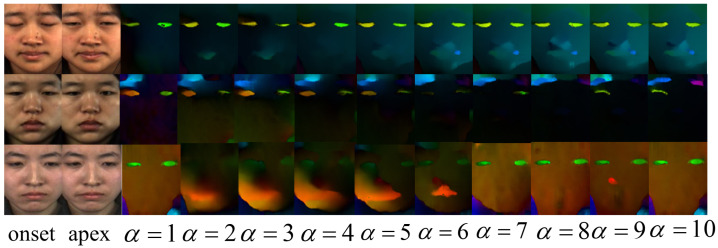
The onset and apex frames of micro-expressions are amplified at different levels, and then the optical flow information is extracted using deep learning, where α represents the magnification factor.

To enhance the differences in micro-expressions, we propose the ARTNet to amplify micro-expressions. Our approach consists of three main steps. The first step is dedicated to amplifying the amplitude of motion of micro-expressions between the onset frame and the apex frame. Here, micro-expressions are initially scaled up by an appropriate factor, followed by the extraction of their optical flow information. In the next part, a deep learning approach is used to compute the optical flow information between the onset frame and the apex frame of the micro-expressions. As illustrated in Figure 1, motion amplification reinforces the small changes of the human face in micro-expressions, which can have an important impact on the improvement of MER. Finally, we focus on integrating both local fine-grained and global coarse-grained interactions at various image scales.

In summary, the key contributions of our work can be summarized as follows:

1. We present a novel approach to processing amplified micro-expression features, aiming to enhance the distinction between the micro-expression onset frame and apex frame. This addresses the issue of the limited range of micro-expression movements.
2. We introduce an innovative self-attention mechanism that prioritizes facial local areas over global areas like the eye and lip regions. Since micro-expression recognition heavily relies on local facial muscle movements, our model effectively captures essential features by focusing on specific facial regions using local attention while disregarding irrelevant areas.
3. Through experiments on three datasets, our proposed method demonstrates significant improvements over previous approaches, showcasing its effectiveness and superiority in micro-expression recognition tasks.

## 2. Related Work

Traditional MER methods rely heavily on appearance-based features [7]. One method is the local binary pattern from three orthogonal planes (LBP-TOP) [8], which merges spatial and temporal dimensions by using the histogram of the local binary pattern (LBP). To capture textures in all directions and reduce redundant features, the Hot Wheel Pattern using three orthogonal planes has been proposed [9]. A variant of LBP with six intersection points has also been suggested [10]. Beyond appearance-based features, motion characteristics of micro-expressions are also critical for recognition. Some researchers have employed the optical flow technique to represent micro-expressive movements. Liong et al. introduced a dual-weighted method called Bi-WOOF, which assigns weights to histograms of directed optical flow by considering both magnitude and optical strain values [11]. By extracting the optical flow [12] between two frames and inputting it into a CNN, computational load is significantly reduced. This article primarily focuses on selecting onset and apex frames in a video clip, extracting the optical flow field between them, and demonstrating that the information in the apex frames effectively characterizes micro-expressions as a whole. Subsequent research has further explored the analysis of micro-expressions by comparing onset and apex frames, leading to the development of models such as EMRNet [13], STSTNet, and DSSN [14]. These models leverage optical flow information as input to 2D CNNs, reducing computational complexity while preserving key features [15,16]. Since micro-expressions are characterized by their brief duration and subtle intensity, exploring methods to enhance their motion range remains a valuable area of study. Motion magnification techniques, such as Eulerian Video Magnification, were proposed by HY Wu. Furthermore, Li Y pioneered the use of deep learning for end-to-end motion amplification, autonomously learning filters from data without hand-designed features. Once features are extracted, they are used as input for classification, which can involve either traditional models [17,18,19] or deep learning-based models. The scaled dot-product attention mechanism, originally introduced in the transformer model, has become a fundamental technique in Natural Language Processing (NLP) tasks. Expanding on this concept, the Vision Transformer (ViT) [20] extends the transformer architecture to computer vision tasks. In the realm of image classification, ViT applies the transformer to non-overlapping blocks of the image. Various powerful versions of Visual Transformers [21,22,23] have been rapidly evolving to meet the demands of image and video classification tasks.

Despite the remarkable advancements in deep learning-based MER methods, a common challenge remains the limited amplitude of micro-expression movements captured by traditional optical flow extraction techniques. In order to address this issue, we introduce a novel network called ARTNet, which amplifies micro-expression movements prior to optical flow extraction. Additionally, we implement an adaptive network that emphasizes different facial regions based on individual characteristics.

## 3. ARTNet

**Figure 2:**
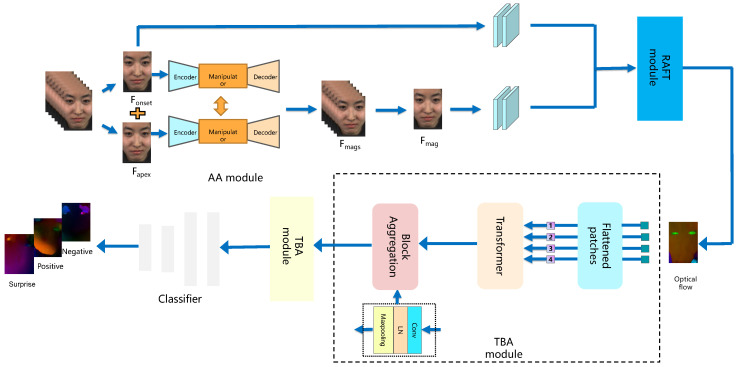
The proposed ARTNet consists of three main modules: the AA module, the RAFT module, and the TBA module.

As illustrated in Figure 2, the proposed ARTNet consists of three main components: the Action Amplification Module (AA module), the Recurrent All-Pairs Field Transforms (RAFT module), and the Transformer and Block Aggregation Module (TBA module). The AA module selects the onset and apex frames of a micro-expression and applies motion amplification at an appropriate level. In the RAFT module, deep learning techniques are employed to extract optical flow information between the onset frame and the magnified frame, effectively capturing motion changes during the occurrence of the micro-expression. The TBA module incorporates a self-attention mechanism within the transformer layer of the low-level network to capture intricate details and features. Block aggregation processes combine smaller image blocks into larger ones to facilitate interactions and extract coarse-grained features. The resulting feature maps are then input into an MLP block for micro-expression classification. This modular and hierarchical design of the ARTNet model effectively extracts and integrates features at different scales and levels of granularity, ultimately enhancing micro-expression recognition performance.

### 3.1. AA Module

Motion magnification is a valuable technique for enhancing subtle movements that may not be easily visible to the human eye but are nonetheless significant for analysis. According to the definition provided by Wadhwa et al. [24] regarding motion magnification, a solitary frame within a sequential video can be characterized as follows:

(1) I(x,t)=f(x+δ(x,t)).

where I(x,t) represents the image at position *x* and moment *t* of the luminance value, and δ(x,t) represents the motion deviation. The goal is to generate the amplified image $\dot{I}\left(x,t\right)$. This can be expressed as

(2) $$\dot{I}\left(x,t\right)=f\left(x+\left(1+\alpha \right)\delta \left(x,t\right)\right).$$

where α is the magnification factor.

Traditional motion amplification techniques primarily depend on optical and physical models, which present three significant limitations in enhancing subtle movements, such as in micro-expression analysis. First, these methods are highly sensitive to environmental noise; while amplifying target motion, they inadvertently enhance high-frequency noise, resulting in a significant reduction in the signal-to-noise ratio. Second, their algorithmic design typically assumes static backgrounds and simple scenes, making it difficult to distinguish genuine motion signals from background interference in dynamic or complex environments, ultimately reducing extraction accuracy. Lastly, traditional methods like Eulerian amplification are constrained by linear approximation theory, leading to theoretical upper limits on amplification coefficients that are inadequate for addressing nonlinear motion features. Collectively, these limitations restrict the practicality and reliability of traditional approaches in complex scenarios.

In the actual process, we use this pre-trained AA module [25] to zoom in on the motion between the onset and apex frames of a micro-expression by a factor of $\alpha_{1}$ to $\alpha_{m}$. This can be formulated as

(3) $$I_{aa}^{\alpha_{m}}=A\left(I_{onset},\alpha_{m},I_{apex}\right).$$

where $I_{onset}$ is the onset frame of a micro-expression. $\alpha_{m}$ stands for magnifying power. $I_{apex}$ is the apex frame of a micro-expression. $I_{aa}^{\alpha_{m}}$ is using the AA model to zoom in on the motion between $I_{onset}$ and $I_{apex}$ by a factor of $\alpha_{m}$ to produce the enlarged picture. A(•) is the AA module.

**Figure 3:**
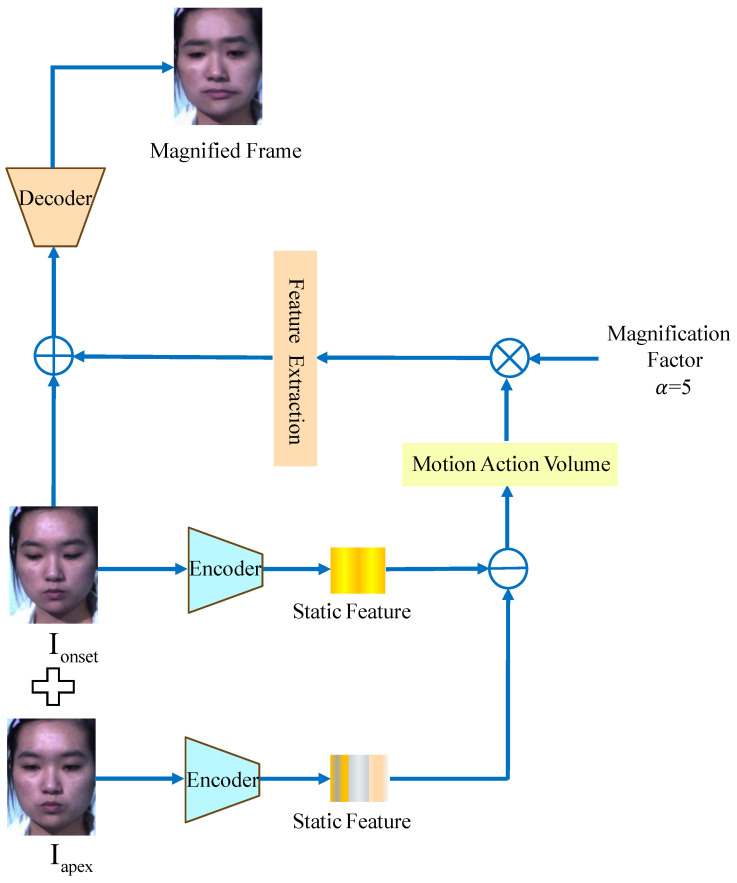
Data from the micro-expression dataset after processing with the AA module.

As illustrated in Figure 3, the AA module begins with the encoder extracting static features from both the onset and apex frames of the micro-expressions. Subsequently, the static information from the onset frame is subtracted from that of the apex frame to derive the motion information. This motion information undergoes operations such as convolution and the application of an activation function. Following this, an amplification factor is applied to enhance the motion information, after which feature extraction is performed on the amplified data, which is then added back to the onset frame. Finally, the decoder processes this combined information to generate the amplified frame. Notably, the motion changes between the amplified frame and the onset frame are more pronounced than those observed between the apex frame and the onset frame. The figure provides a clear illustration of the significant changes in the mouth region between the amplified frame and the onset frame.

### 3.2. RAFT Module

Researchers have highlighted the significance of optical flow features in motion estimation and have developed several enhanced techniques for extracting optical flow [11,26,27]. Optical flow represents the projection of an object’s movement in three-dimensional space onto a two-dimensional image plane. It is created by the relative velocity of the object and the camera, indicating the direction and speed of the object’s image pixels over a brief period. The optical flow approach is grounded on three key assumptions. The first assumption is constant brightness, which states that the pixel brightness of an object in an image remains unchanged between consecutive frames. The second assumption is short distance or short-time motion, where the time interval between adjacent frames is brief enough for the object to move minimally. The third assumption is spatial consistency, indicating that neighboring pixels exhibit similar motion. h(x,y,t) is the pixel value of the pixel point (x,y) at the moment of *t*. Then, according to the first two assumptions, it can be obtained as

(4) h(x,y,t)=h(x+dx,y+dy,t+dt).

Equation (4) passes through a first-order Taylor expansion, which yields

(5) $$h\left(x+dx,y+dy,t+dt\right)=h\left(x,y,t\right)+\frac{\partial h}{\partial x}dx+\frac{\partial h}{\partial y}dy+\frac{\partial h}{\partial t}dt.$$

From Equations (4) and (5), it can be inferred that

(6) $$\frac{\partial h}{\partial t}=-\frac{\partial h}{\partial x}\frac{dx}{dt}-\frac{\partial h}{\partial y}\frac{dy}{dt}.$$

The RAFT [28] algorithm, developed in the era of deep learning, aims to estimate optical flow by iteratively updating the flow field using GRU cyclic cells. This mimics the optimization process seen in traditional approaches. The RAFT network is composed of three main layers: the feature coding layer, the feature association layer, and the cyclic update operator. The feature coding layer extracts pixel-by-pixel features, while the feature association layer generates 4D correlation information for all pixels. The convergence layer produces lower-resolution correlation information. The cyclic update operator, based on GRU principles, uses 4D correlation information to iteratively update the optical flow field starting from an initial zero setting.

In the actual process, we utilize this pre-trained RAFT module to compute the optical flow map between the micro-expression’s onset frame and the magnified frame. This can be represented as

(7) $${\dot{I}}_{\mathrm{raft}\;}^{\alpha_{m}}=R\left(I_{\mathrm{onset}\;},I_{\mathrm{aa}\;}^{\alpha_{m}}\right).$$

where $I_{\mathrm{onset}}$ is the onset frame of a micro-expression. $\alpha_{m}$ denotes magnifying power. $I_{\mathrm{aa}}^{\alpha_{m}}$ is an image generated through the application of the AA module. We use the RAFT module to calculate the optical flow between $I_{\mathrm{onset}}$ and $I_{\mathrm{apex}}$ to obtain ${\dot{I}}_{\mathrm{raft}}^{\alpha_{m}}$. R(•) is the RAFT module.

### 3.3. TBA Module

The TBA module is composed of transformer layers and block aggregation layers, with each transformer independently processing the features of image blocks. Initially, horizontal and vertical optical flow motion maps are computed between the onset and apex frames of micro-expressions to effectively represent facial motion. This foundational step lays the groundwork for subsequent feature analysis, enabling the model to capture subtle dynamic changes in facial expressions. Next, the TBA module utilizes the multi-head attention mechanism of the transformer to model facial features. This approach accurately analyzes the dependencies among key facial regions, such as the lips, eyes, and nose, thereby improving the precision of feature extraction. Additionally, the block aggregation layer facilitates adaptive fusion of spatiotemporal features through a dynamic weight allocation strategy. In the preceding network, the self-attention mechanism of the transformer captures fine-grained features, while the block aggregation layer consolidates small image blocks into larger ones, allowing interaction between different blocks and the extraction of coarse-grained features. Lastly, the MLP layer within the model is employed for classifying the final feature map into distinct micro-expression categories.

#### 3.3.1. Transformer Layer

The architecture consists of multiple transformer layers, where each layer utilizes visual tokens and key point tokens as input. Within each block, there is a Layer Normalization (LN) operation followed by a multi-head self-attention (MHA) module and a Feedforward Neural Network (FFN) module. MHA operation is structured in the following way:

(8) $$MHA\left(X^{l}\right)=\mathrm{concat}\left(\mathrm{soft}\;\max\left(\frac{Q\cdot K^{T}}{\sqrt{d_{k}}}\right)\cdot V\right)\cdot M.$$

where *Q*, *K*, and $V\in R^{S\times d}$. During the transformation process of the transformer layer, *S* represents the sequence length and *d* is the dimension of the inputs. $M\in R^{d\times d}$ refers to the combination of h attention heads. $X^{l}$ indicates the output tensor of *l*, which is the transformer layer. $\frac{1}{\sqrt{d_{k}}}$ is the scaling factor.

LN will be applied in each block as follows:

(9) $$LN\left(x\right)=\frac{x-\mu }{\delta }o\lambda +\beta .$$

where μ is the mean of features. δ is standard deviation of the features. *o* is the element-wise dot. λ and β are learnable parameters.

The FFN layer can be expressed as

(10) $$FFN\left(x\right)=\max\left(0,xW_{1}+b_{1}\right)W_{2}+b_{2}.$$

#### 3.3.2. Block Aggregation

Our ARTNet’s block aggregation function incorporates a hierarchical structure and feature fusion to capture multi-scale information, facilitating robust analysis of visual data across different scales. Additionally, our model leverages local attention for individual image blocks, leading to significant enhancements in performance. The accurate recognition of micro-expressions is heavily reliant on detecting localized facial muscle motion regions. By employing local attention and focusing on specific facial regions, our model effectively captures essential features for accurately inferring micro-expression states.

In the ARTNet, we enable the exchange of local and global features. Low-level block aggregation focuses on local facial areas to extract intricate details of facial dynamics, while high-level block aggregation facilitates global information exchange, capturing broader facial expressions. The block aggregation process in the ARTNet consists of several steps: it begins with a 3 × 3 convolutional layer, followed by Layer Normalization (LN) and a 3 × 3 max pooling operation. The facial optical flow map is initially divided into 16 blocks (4 × 4 feature maps). The first convolution combines features from these areas, reducing the block size to 2 × 2. A second convolution then exchanges information between the areas, further reducing the size to 1 × 1. This process extracts complete optical feature maps, which are input into an MLP layer for micro-expression classification. This hierarchical approach captures and combines features at different levels of detail, enhancing micro-expression recognition.

### 3.4. Loss Function

In this work, we utilize the cross-entropy loss function to train our model.

(11) $$L=\sum_{i}\left(-w_{i}\log\left(p_{t}^{i}\right)\right).$$

(12) $$p_{t}=p_{c}^{y}{\left(1-p_{c}\right)}^{1-y}.$$

where $w_{i}$ is the weight assigned to each sample in the dataset, and *y* represents the ground truth label, and $y_{i}\in \left\{0,1\right\}$.

## 4. Experiments

### 4.1. Datasets

We conducted experiments using three datasets: CASME II [29], SMIC [30], and SAMM [31]. The SAMM datasets do not pre-process for face cropping. In order to reduce the interference of the background, we use multi-task cascaded convolutional networks (MTCNNs) [32] to conduct preprocessing for face cropping. Especially, the SAMM and CASME II benchmark datasets include labeled onset, apex, and offset frames for each micro-expression sequence. However, the SMIC does not label the apex frame. We try to use intermediate position frame instead of apex frames. This is because we found that most of the apex frames are located in the middle part of the micro-expression video.

### 4.2. Evaluation Metrics

To minimize the influence of specific subjects, we utilize the leave-one-subject-out (LOSO) cross-validation approach. In each iteration, samples from one subject are reserved for testing, while samples from the remaining subjects are used for training. This process is repeated *S* times, where *S* = 27 in our experiments. We assess performance using the unweighted F1-score (UF1) and unweighted average recall (UAR). UF1 calculates the average F1-score across all classes, providing an overall measure of classification accuracy that is not affected by class imbalances. UAR computes the average recall across all classes without considering class weights, with UF1 defined as the average F1 across all $n_{c}$ classes.

In order to eliminate the impact of specific subjects during the process, we employ the leave-one-subject-out (LOSO) cross-validation approach. During each iteration, samples belonging to a single subject were kept aside for testing purposes, while all the remaining samples from other subjects were used for training the model. The experiment was repeated *S* times, where *S* represents the total number of subjects in the dataset. *S* is 27 in our experiments. To assess the performance, the unweighted F1-score (UF1) and unweighted average recall (UAR) metrics are employed. UF1 calculates the average F1-score across all classes, providing an overall measure of classification accuracy that is not influenced by class imbalances. UAR computes the average recall across all classes without considering class weights. UF1 is calculated as the average F1 across all classes $n_{c}$ as

(13) $$UF1=\frac{1}{n_{c}}\sum_{j}F1_{j}.$$

where $F1_{j}=\frac{2\sum_{s=1}^{S}TP_{j}^{s}}{2\sum_{s=1}^{S}TP_{j}^{s}+\sum_{s=1}^{S}FP_{j}^{s}+\sum_{s=1}^{S}FN_{j}^{s}}$ is the F1 for the class. UAR is defined as the average recall as

(14) UAR=1nc∑jRecallj.

where Recallj=∑s=1STPjs∑s=1SFPjs+∑s=1SFNjs. $TP_{j}^{s}$, $FP_{j}^{s}$, and $FN_{j}^{s}$ are true positives, false positives, and false negatives for *j* class of the *s* subject.

### 4.3. Implementation Details

The AA module is applied to focus on magnifying the motion between the onset and apex frames. Distinct values of $\alpha_{m}$ are necessary for various datasets. For the CASME II dataset, $\alpha_{m}$ is 3. For the SAMM dataset, $\alpha_{m}$ is 2. For the SMIC dataset, $\alpha_{m}$ is 7. Subsequently, the RAFT module calculates the optical flow between the onset and magnified frames of the micro-expression. The MTCNN is used to extract face landmark coordinates from the magnified images, which are essential for accurately localizing specific facial regions. Finally, the TBA module is employed to capture the relevant facial muscle movements related to micro-expressions, and these features are input to an MLP for classification.

Our experiments are conducted on a system with an Intel Core™ i7-9700 CPU, 16 GB RAM, and a GIGABYTE 3090Ti GPU using Ubuntu 20.04 and PyTorch 1.7. We optimize the model with the Adam optimizer, a learning rate of 0.00005, 800 epochs, and a batch size of 16.

### 4.4. Magnification Setting

Facial muscle movements become more noticeable at higher amplification levels, offering important visual cues for micro-expression recognition. However, excessive amplification can lead to facial distortion. To find an optimal balance between enhancing visibility and maintaining facial authenticity, we will conduct comparative experiments using the CASME II, SMIC, and SAMM datasets. In this study, we utilize UF1 and UAR metrics to systematically assess the effects of different amplification factors on micro-expression recognition performance.

As shown in Table 1, Table 2 and Table 3, with $\alpha_{m}$ increasing, UF1 and UAR show large increases in these datasets. In the CASME II dataset, UF1 and UAR reach their maximum values when $\alpha_{m}$ is 3, while, in the SMIC dataset, they peak at $\alpha_{m}$ = 7, and, in the SAMM dataset, the maximum values occur at $\alpha_{m}$ = 2. Compared to the optical flow of micro-expressions without magnification, both UF1 and UAR are notably improved, which reflects the effectiveness of the magnification strategy.

### 4.5. Comparison with the State of the Art

Conventional expression recognition techniques often use local binary patterns from three orthogonal planes (LBP-TOP) [8], which is effective for this purpose. LBP-TOP enhances micro-expression recognition by employing local binary patterns with six intersection points (LBP-SIP) for facial feature extraction. Bi-WOOF [11] utilizes the Bi-Weighted Optical Flow method to extract key facial characteristics from apex images, effectively capturing relevant facial motions and emphasizing critical details for micro-expression identification. To address cross-database challenges, Dual-Inception [33] introduces two inception networks that extract horizontal and vertical features from optical flow maps. STSTNet [34] uses a model with three shallow CNN layers to obtain high-level discriminative representations for micro-expression emotion classification. FeatRef [35] operates in two phases: first, it extracts horizontal and vertical muscle motion features using separate inception networks and then combines these features for classification through three attention-based networks. Finally, the classification branch integrates key features from the inception module to identify micro-expressions.

As shown in Table 4, both UF1 and UAR of our ARTNet having high performance on all three datasets proves that the ARTNet can adapt to more complex situations. On the CASME II, both UF1 and UAR of our framework ARTNet are higher than 0.7900. Especially, on the SAMM, the ARTNet improves by 3.35% and 1.30% on UF1 and UAR over TFT [36]. On the SMIC, the ARTNet improves by 3.10% and 5.12% on UF1 and UAR over TFT. This indicates that the ARTNet performs excellently in handling diverse micro-expression samples.

## 5. Conclusions

Due to their short duration and low amplitude of movement, micro-expressions are challenging to characterize as valid inputs for the network. To address this, we introduce a new adaptive network designed for action amplification in micro-expression recognition. This network comprises three key modules: the AA module, the RAFT module, and the TBA module. The AA module utilizes deep learning techniques to enhance the motion of micro-expression frames, specifically focusing on the onset and apex frames. Similarly, the RAFT module employs deep learning techniques to calculate the optical flow of micro-expressions at these critical frames. The TBA module further emphasizes essential components within the derived optical flow information, ultimately enhancing overall performance.

While this work provides an in-depth exploration of micro-expression recognition and proposes effective methods, several important considerations warrant attention for future research. First, the current reliance on the CASME II, SMIC, and SAMM datasets should be broadened to include samples from various age groups, as well as diverse social contexts and emotional states. Such an expansion would significantly enhance the model’s generalization capabilities. Furthermore, the high complexity of the ARTNet may hinder its practicality in real-time applications. Therefore, incorporating model compression techniques, such as pruning and quantization, is recommended to mitigate this complexity and facilitate more effective real-time implementation.

## Figures and Tables

**Table 1:** Assessment of various magnification levels on the CASME II dataset was conducted to evaluate their impact and effectiveness.

$\alpha_{m}$	1	2	3	4	5	6	7	8
UF1	0.7398	0.7545	0.8380	0.7697	0.7693	0.7716	0.6915	0.7816
UAR	0.6858	0.7178	0.7983	0.7222	0.7160	0.7337	0.6594	0.7370

**Table 2:** Assessment of various magnification levels on the SMIC dataset was conducted to evaluate their impact and effectiveness.

$\alpha_{m}$	1	2	3	4	5	6	7	8
UF1	0.7281	0.6479	0.6961	0.6944	0.7217	0.7300	0.7720	0.7137
UAR	0.7660	0.6470	0.6886	0.6932	0.7172	0.7304	0.7692	0.7105

**Table 3:** Assessment of various magnification levels on the SAMM dataset was conducted to evaluate their impact and effectiveness.

$\alpha_{m}$	1	2	3	4	5	6	7	8
UF1	0.6214	0.7425	0.6534	0.6623	0.7257	0.6997	0.5506	0.7181
UAR	0.5591	0.6690	0.5942	0.5983	0.6684	0.6480	0.5154	0.6474

**Table 4:** The UF1 and UAR of different methods were evaluated using the leave-one-subject-out (LOSO) protocol on three datasets.

Database	CASME II		SAMM		SMIC	
	UF1	UAR	UF1	UAR	UF1	UAR
LBP-TOP [8]	0.7026	0.7429	0.3954	0.4102	0.2000	0.5280
CapsuleNet [16]	0.7068	0.7018	0.6209	0.5989	0.5820	0.5877
Bi-WOOF [11]	0.7805	0.8026	0.5211	0.5139	0.5727	0.5829
GoogLeNet [37]	0.5989	0.6414	0.5124	0.5992	0.5123	0.5511
VGG16 [38]	0.8166	0.8202	0.4870	0.4793	0.5800	0.5964
Dual-Inception [33]	0.8621	0.8560	0.5868	0.5663	0.6645	0.6726
STSTNet [34]	0.8382	0.8686	0.6588	0.6810	0.6801	0.7013
FeatRef [35]	0.8915	0.8873	0.7372	0.7155	0.7011	0.7083
SLSTT-LSTM [39]	0.9010	0.8850	0.7150	0.6430	0.7400	0.7200
TFT [36]	0.9070	0.9090	0.7090	0.6560	0.7410	0.7180
ARTNet	0.8380	0.7983	0.7425	0.6690	0.7720	0.7692

## Data Availability

The original contributions presented in this study are included in the article. Further inquiries can be directed to the corresponding author.
